# Brain organoid data synthesis and evaluation

**DOI:** 10.3389/fnins.2023.1220172

**Published:** 2023-08-15

**Authors:** Clara Brémond-Martin, Camille Simon-Chane, Cédric Clouchoux, Aymeric Histace

**Affiliations:** ^1^ETIS Laboratory UMR 8051 (CY Cergy Paris Université, ENSEA, CNRS), Cergy, France; ^2^Witsee, Neoxia, Paris, France

**Keywords:** psychovisual, metric, validation, brain organoid, AAE

## Abstract

**Introduction:**

Datasets containing only few images are common in the biomedical field. This poses a global challenge for the development of robust deep-learning analysis tools, which require a large number of images. Generative Adversarial Networks (GANs) are an increasingly used solution to expand small datasets, specifically in the biomedical domain. However, the validation of synthetic images by metrics is still controversial and psychovisual evaluations are time consuming.

**Methods:**

We augment a small brain organoid bright-field database of 40 images using several GAN optimizations. We compare these synthetic images to the original dataset using similitude metrcis and we perform an psychovisual evaluation of the 240 images generated. Eight biological experts labeled the full dataset (280 images) as syntetic or natural using a custom-built software. We calculate the error rate per loss optimization as well as the hesitation time. We then compare these results to those provided by the similarity metrics. We test the psychovalidated images in a training step of a segmentation task.

**Results and discussion:**

Generated images are considered as natural as the original dataset, with no increase of the hesitation time by experts. Experts are particularly misled by perceptual and Wasserstein loss optimization. These optimizations render the most qualitative and similar images according to metrics to the original dataset. We do not observe a strong correlation but links between some metrics and psychovisual decision according to the kind of generation. Particular Blur metric combinations could maybe replace the psychovisual evaluation. Segmentation task which use the most psychovalidated images are the most accurate.

## 1. Introduction

The scarcity of public datasets of annotated biomedical images remains an unresolved bottleneck to develop specialized and robust analysis tools. Often, research groups do not share experimental data for privacy reasons. The high costs of equipment, long acquisition times, and necessary in-depth expertise can be a brake to acquisitions by other teams (Chakradhar, 2016). To benefit from the advances in deep-learning (DL) for automated image analysis, large training datasets are necessary. Moreover, original dataset constraints create a problem of class imbalance with deep learning training procedures. These problems are emphasized with small sets, reduced to a few images (Tajbakhsh et al., 2016).

Data augmentation is widely used in the biomedical domain to increase the size of image datasets (Singh and Raza, 2021). While classical data augmentation, based on transformations (flip-flops, rotation, whitening, etc.), does not increase the diversity of the dataset, a solution widely provided in the biomedical field is the use of Generative Adversarial Networks (GAN) which produce new synthetic images from natural ones (Yi et al., 2019; Lan et al., 2020).

GANs are unsupervised deep-based architectures composed of generator and a discriminator. The generator aims at creating visually realistic and natural images while the discriminator tries to decipher whether the result is generated (Goodfellow et al., 2014). Both networks are trained simultaneously with the same loss function. Since its first creation, multiple GAN architecture variations have been proposed to generate and extend biomedical datasets (Lan et al., 2020; Fernandez et al., 2021; Chen et al., 2022). However, the validation of these synthetic images remains a challenge Alqahtani et al. (2019). Two main evaluating methods are existing the non-automated based upon psychophysics methods in perceptual psychology, and automated methods based upon metrics (Salimans et al., 2016a; Zhou et al., 2019). Psychovisual evaluation is a time consuming gold standard which requires many subjects to reduce its' intrinsic subjectivity. On the other hand, there is no commonly approved specific metric to evaluate whether GAN-generated synthetic images can be considered as natural. The use of common metrics is also controversial (Borji, 2019).

We consider the case of brain organoids, three dimensional cultures differentiated from pluripotent stem cells (Lancaster et al., 2013). After a neural induction, brain organoids present cell communications, organized tissues and an organization similar to the brain with various regions such as neuro-epithelial zones (Kelava and Lancaster, 2016). They complement *in vivo* brain models to follow physiological and pathological brain development. However, brain organoids suffer from batch syndrome: in the same culture environment they do not innately develop comparable morphologies (Lancaster et al., 2013).

There are no specific tools to study the development of brain organoids. Though it may seem natural to implement machine learning algorithms to aid this task, few brain organoid image datasets are publicly available. Between january 2018 and june 2020 for instance only six over 457 articles in the brain organoid field let the image datasets in openaccess and only one concerns brain organoids in bright-field (Brémond Martin et al., 2021). The emergence of brain organoids has created a new field, few laboratories have the knowledge and experience necessary to grow these cultures. Pandemic restrictions have further limited the possibility for several teams to grow and image such culture over the past few years.

The largest public brain-organoid image dataset we know of contains 40 images (Gomez-Giro et al., 2019). Data augmentation solutions have already been used to increase the size and diversity of this brain organoid bright-field dataset. An adversarial autoencoder (AAE) seems the architecture the most suited to augment images of brain organoid bright-field acquisition (Brémond Martin et al., 2022a). This AAE differs from the original GAN architecture by the input given to the encoding part (original images) and its generative network containing an auto-encoder-decoder framework (Goodfellow et al., 2014; Makhzani et al., 2016). The encoder learns to convert the data distribution to the prior distribution, while the decoder learns a deep generative model that maps the imposed prior to the data distribution thanks to a latent space (Makhzani et al., 2016). As is typical with AAEs, the images generated are visibly blurry.

To improve the sharpness during the generation, we test various loss functions to improve the adversarial network (Brémond Martin et al., 2022a). However, these results are based upon metric calculation and a dimensional reduction to compare all feature images (original and generated with each optimization) in the same statistical space. Indeed some metrics may not been suited to identify the naturality of an image as they are originally created to test the similitude or the quality of images (Borji, 2019; Brémond Martin et al., 2022a). In our previous contribution we observe the data augmentation strategy based upon AAE loss optimizations used during the training step of a DL segmentation algorithm improve the quality of the shape extraction of brain organoids (Brémond Martin et al., 2022a). The first raised question is does these images seems as natural as the original images to furnish a better segmentation quality compared to a result issue from classic data augmentation strategies? Another fundamental issue remains unresolved: do these synthetic images seem natural to a biological expert point of views as for metric(s)? Psychovisual evaluations have been already made on others bright-field cell synthetic cell generation (Malm et al., 2015). This evaluation is an important step for the validation of a particular generative model of images. Thus the selected images as natural by Human Biological experts could maybe help to train deep based segmentation methods and characterize their development but with now a double psychovisual-metric validation.

We propose to evaluate the synthetic images generated by an AAE (Brémond Martin et al., 2022a) using both with similarity metrics and biological experts. The purpose of this article is to give the lacking non-automated psychovisual evaluation of the synthetic images which is a new contribution compared to our previous contribution which focus on automated metric based and statistical strategies in order to understand if the naturality of these images could explain the segmentation results (Brémond Martin et al., 2022a). The second original part of the work is to find a metric combination which may replace or complete the psychovisual non-automated evaluation. Related work is presented in the following section. Section 3 describes the generative network, the metric evaluation and the psychovisual evaluation. Section 4 successively shows the results for the metric evaluation and the pyschovisual evaluation, followed by a cross-analysis of the two evaluation methods. These results are analyzed in the Section 5. Section 6 sums up the main contributions of this paper.

## 2. Related work

The first generative adversarial network, proposed by Goodfellow et al. (2014), is constituted by two connected networks: the generative model (*GM*) maps the images into the space (*z*) by an objective function (*F*); the discriminative model (*DM*) determines the probability for which a point from *z* belongs to the original dataset (*o*) or to the generated dataset (*g*). Training *F* increases the probability that the data synthesized is attributed to *o*. The probability of correct sample labeling (belonging to generated *g* or original *o*) is maximized by *D*. Simultaneously, *GM* is trained to leverage the discriminator function expressed by:


(1) $$\begin{array}{l} \underset{GM}{\min}\underset{DM}{\max}F\left(DM,GM\right)=E_{o}p_{\text{data}}\left[logD_{o}\right]+E_{g}p_{g}\left[log\left(1-D\left(G_{z}\right)\right)\right]. \end{array}$$

An overview of various GAN architectures used for medical imaging is given by Yi et al. (2019). Previous work compares five GAN architectures commonly used for biological datasets to find the best suited network to increase a small database of bright-field images of organoids (Brémond Martin et al., 2022a): the original GAN implementation (Goodfellow et al., 2014); CGAN gives to the generator input the correct label (physiological or pathological) (Mahmood et al., 2018); DCGAN by Radford et al. (2015) is constituted by a convolutional neural networks instead of the generator; INFOGAN uses the generated images at an epoch to train the subsequent (Hu et al., 2019); AAE by Makhzani et al. (2016) uses an auto-encoder as a generator. This work evaluates the generated images using metrics.

As already reported in the literature by Lan et al. (2020), GAN and CGAN produce mode collapse with such a small dataset. In addion, GAN produces a white imprint around the shape of the organoid. DCGAN and INFOGAN generate a divergent background which makes the images difficult to exploit. This is probably due to the high variability of the small dataset, as mentioned in Lan et al. (2020). Only AAE produces images of similar quality to the orignal and seems the best architecture to generate images with a few input. However, AAE generates blurry images and the background differs from the light-to-black gradient present in many bright-field images.

Noise-injection during the AAE generation was studied to improve the background generation with satisfying results in Brémond Martin et al. (2022b). In this paper, we further explore the effect of loss optimization on the AAE architecture to improve the sharpness of the generated images. The results are evaluated by an automatic approach based on metrics and by a psychovisual study. The last part aims at giving an application of such psychovalidated images in a segmentation task.

## 3. Methods

In this section, we first present the generative methods and optimizations used. We follow by a description of the metric and psychovisual evaluations including the experimental setup, datasets and experts. We present the analysis methodologies of the neurobiologist decisions and the comparison of psychovisual evaluations with metric calculations. We finish by giving an asset of the importance of psychovalidated images in an augmented dataset strategy for a segmentation task.

### 3.1. Generative adversarial networks

#### 3.1.1. Original images

Our dataset is composed of 40 microscope acquisitions and 240 synthetic images created by a AAE loss optimizations. “Original” images are the 40 bright-field brain organoid acquisitions provided by Gomez-Giro et al. (2019). This dataset is made of image of 20 physiological and 20 pathological organoids acquired over three days on the same apparatus. These input images (1, 088 × 1, 388 pixels) are cropped and scaled to 250 × 250 pixels, maintaining their original proportions. A scale factor of 4 is chosen so the scripts can run in a reasonable amount of time without downgrading the input image quality too much.

#### 3.1.2. Loss optimizations

The images generated by the AAE architecture we use are somewhat blurry. To overcome this phenomenon we study how the discriminator loss can influence the quality of the image generation. We consider six losses: the Binary Cross Entropy (BCE) which is most commonly used in GANs and five other losses which are specifically known to improve the contrast or sharpness of the generated images.

BCE is the most commonly used loss for GANs and the baseline of this work. It is calculated by:


(2) $$\begin{array}{l} \text{BCE}=-\frac{1}{n}\overset{n}{\underset{i=1}{\sum }}\left(y_{i}\left(\log\left(y_{i}^{\prime }\right)\right)\right)-\left(\left(1-y_{i}\right)\left(\log\left(1-y_{i}^{\prime }\right)\right)\right) \end{array}$$

with *y* the real image tensor and *y*′ the predicted ones (Makhzani et al., 2016) and *n* the number of training.

Summing the L1 norm to the BCE is reported to reduce over-fitting (Wargnier-Dauchelle et al., 2019). We hypothesize this norm could improve the quality of the generation as reported in image restoration tasks which does not over-penalize large errors (Zhao et al., 2017).


(3) $$\begin{array}{l} \text{L}1=\frac{1}{n}\overset{n}{\underset{i=1}{\sum }}|y_{i}-y_{i}^{\prime }{|}_{1} \end{array}$$

(4) $$\begin{array}{l} {\text{BCE}}_{\text{L1}}=\text{BCE}+\alpha \text{L}1. \end{array}$$

α is set to 10^−4^, as in the original paper.

The least square loss (LS) is reported by Mao et al. (2017) to avoid gradient vanishing in the learning process step resulting in better quality images:


(5) $$\begin{array}{l} \text{LS}=\frac{1}{n}\overset{n}{\underset{i=1}{\sum }}{\left(y_{i}-y_{i}^{\prime }\right)}^{2} \end{array}$$

A Poisson loss is used in Wargnier-Dauchelle et al. (2019) to improve the sensitivity of a segmentation task:


(6) $$\begin{array}{l} L_{Poisson}=\frac{1}{n}\overset{n}{\underset{i=1}{\sum }}\left(y_{i}^{\prime }-y_{i}\right)\log\left(y_{i}^{\prime }+\epsilon \right) \end{array}$$

where ϵ is a regularization term set to 0.25.

The DeblurGAN was developed to unblur images using the Wasserstein loss (Kupyn et al., 2018). Since we are also interested in deblurring the output images, we have tested this loss with the proposed AAE where (*P*(*y, y*′)) is the joint distributions of *y* and *y*′ for which the distributions are equal to *Py* and *Py*′, and p(y,y') the proportion of *y* or y' to move to have *Py* = *py*′:


(7) $$\begin{array}{l} Wass\left(P\left(y,y^{\prime }\right)\right)=\overset{n}{\underset{i=1}{\sum }}\underset{p\left(P\left(y,y^{\prime }\right)\right)}{\inf}E_{yi,yi^{\prime }}\delta p\left(\left||yi-yi^{\prime }|\right|\right) \end{array}$$

However, we do not apply a l2 content loss such as in Kupyn et al. (2018) added to the Wasserstein loss, or add a penalty gradient to the Wasserstein loss such as in Gulrajani et al. (2017). Our dataset containing various subclasses (physiological and pathological brain organoid images acquired at three developmental stages), we aim at creating a Normalized Wasserstein loss to avoid imbalanced mixture proportions (Balaji et al., 2019). We apply the L2 normalization on the Wasserstein loss, producing a new loss we call the Perceptual Wasserstein loss for the first time to our knowledge, applied on an AAE architecture:


(8) $$\begin{array}{l} P.Wass\left(P\left(y,y^{\prime }\right)\right)=\overset{n}{\underset{i=1}{\sum }}\sqrt{\underset{p\left(P\left(yi,yi^{\prime }\right)\right)}{\inf}E_{yi,yi^{\prime }}\delta p{\left(\left||yi-yi^{\prime }|\right|\right)}^{2}} \end{array}$$

#### 3.1.3. Training

**Figure 1:**
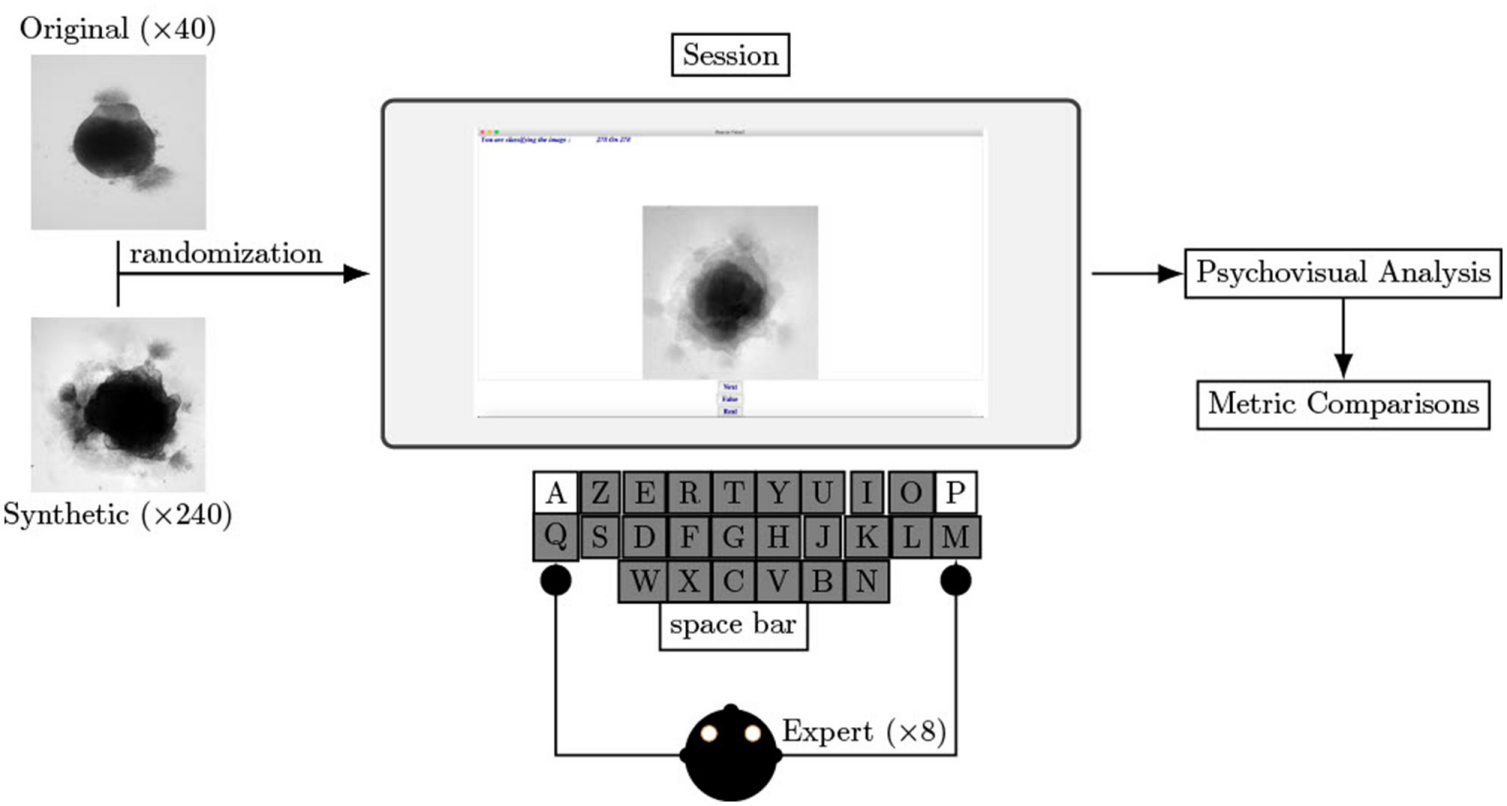
Experimental scheme of AAE supporting data augmentation of cerebral organoids bright-field images. The generator tries to persuade the discriminator that it has generated a true and slightly variable image of input dataset. The discriminator tries to find the true ones. They improve each other by backpropagation, formulated by an objective function based on a loss. Losses variations implemented in this article are symbolized by Δ. Input image is from Gomez-Giro et al. (2019).

Figure 1 shows the global training setup. The 40 original images are used to generate 40 ǎsynthetic images for each architecture (each loss). Input and output images measure 250 × 250 pixels. Training lasts 2,000 epochs for each optimization; this corresponds to the plateau before over-fitting for all loss optimizations.

#### 3.1.4. Resources

The GAN algorithms are developed in Python 3.6 with an Anaconda framework containing the 2.3.1 Keras and 2.1 Tensorflow versions. All scripts are executed on an Intel Core i7-9850H CPU with 2.60 GHz and a NVIDIA Quadro RTX 3000s GPU device.

### 3.2. Metric evaluation

Six metrics are used to compare the similitude of the synthetic images generated by the AAE to the original dataset. A blur metric is used to evaluate the quality of these synthetic images.

The Frechet Inception Distance (FID) is calculated between two groups of images (Heusel et al., 2017). This score tends toward low values when the two groups (original *O* or generated *G* images) are similar, with μ the average value of the pixels of all images of a group, and Σ the covariance matrix of a group:


(9) $$\begin{array}{l} \text{FID}\left(O,G\right)=∥\mu_{O}-\mu_{G}{∥}^{2}{+}^{T}\left(\Sigma_{O}+\Sigma_{G}-2{\left(\Sigma_{O}\Sigma_{G}\right)}^{\frac{1}{2}}\right) \end{array}$$

The Structural Similarity Index (SSIM) is calculated using luminescence, contrast and structure by Wang et al. (2004) between two images *o* and *g* belonging respectively to *O* and *G*.


(10) $$\begin{array}{l} \text{SSIM}\left(o,g\right)=\frac{\left(2\mu_{o}\mu_{g}+c_{1}\right)\left(2\sigma_{og}+c_{2}\right)}{\left(\mu_{o}^{2}+\mu_{g}^{2}+c_{1}\right)\left(\sigma_{o}^{2}+\sigma_{g}^{2}+c_{2}\right)} \end{array}$$

where σ represents the standard deviation, *c*_1_ is a constant that ensures the luminance ratio is always positive when the denominator is equal to 0, and *c*_2_ is an other constant for the contrast stability. The SSIM ranges between 0 (no similitude) and 1 (high similitude).

The Universal Quality Metric (UQM) is based on the calculation of the same parameters as SSIM and was proposed by Wang and Bovik (2002). UQM ranges between 0 and 1 (1 being the highest quality):


(11) $$\begin{array}{l} \text{UQM}\left(o,g\right)=\frac{4\mu_{o}\mu_{g}\mu_{og}}{\left(\mu_{o}^{2}+\mu_{g}^{2}\right)\left(\sigma_{o}^{2}+\sigma_{g}^{2}\right)} \end{array}$$

Entropy-based Mutual Information (MI) measures the correlation between original and generated images and ranges between 0 (no correlation) and 1 (high correlation) (Pluim et al., 2003):


(12) $$\begin{array}{l} \text{MI}\left(o,g\right)=\underset{o\in O}{\sum }\underset{g\in G}{\sum }P\left(o,g\right)log\frac{P\left(o,g\right)}{P\left(o\right)P\left(g\right)} \end{array}$$

where *P*(*o, g*) is the joint distribution of *o* belonging to *O* and *g* from *G*.

The Mean Square Error (MSE) between an original image and a synthetic image is calculated as:


(13) $$\begin{array}{l} \text{MSE}\left(o,g\right)=\frac{1}{mn}\overset{m}{\underset{i=1}{\sum }}\overset{n}{\underset{j=1}{\sum }}{\left(o\left(i,j\right)-g\left(i,j\right)\right)}^{2} \end{array}$$

The Peak Signal to Noise Ratio (PSNR) indicates a high signal power against noise, as used in Jiang et al. (2021). High values correspond to qualitative images. Pixels in images are ranked between 0 and 255, thus the maximum pixel value of an image is noted *max*(*o*) and equals at most 255.


(14) $$\begin{array}{l} \text{PSNR}\left(o,g\right)=20\log\max\left(o\right)-20\log\text{MSE}\left(o,g\right) \end{array}$$

where log denotes the common logarithm.

As a quality metric we calculate the blur index proposed by Tsomko et al. (2008) based on local image variance. A low score corresponds to a sharp image. In the following equation, the image is of size (*m*,*n*), the predictive residues a given image pixel are *p*(*i, j*) and their median *p*′(*i, j*):


(15) $$\begin{array}{l} \text{Blur}=\frac{1}{m\left(n-1\right)}\overset{m}{\underset{i=1}{\sum }}\overset{n-1}{\underset{j=1}{\sum }}{\left[p\left(i,j\right)-p^{\prime }\left(i,j\right)\right]}^{2} \end{array}$$

The FID is designed to compare groups of images. We thus successively compare each group of synthetic images with the original input images. The FID reference range is calculated on the original image developmental stages. The SSIM, UQM, PSNR, MI, and MSE are designed to compare two images. For each group of synthetic images (all six losses) we successively compare every image with each original image and then compute the average of these 40 × 40 values. We also calculate these values on all pairs of original images to compare the results to the original range. The Blur index is calculated on individual images. We store the minimum and maximum value of this index for the original images and the average value per loss for the synthetic images.

### 3.3. Psychovisual evaluation

#### 3.3.1. Dataset of original and synthetic images

The dataset to evaluate contains two classes of images the 40 microscopic acquisitions mentionned in Section 3.1.1 and the 240 synthetic images created by the previous mentioned AAE loss optimizations: binary cross entropy (BCE), binary cross entropy with a L1 normalization (BCE + L1), least squares (LS), Poisson, Wasserstein (Wass.), perceptual Wassertein (P. Wass). The size of these 280 images is 250 × 250 pixels.

#### 3.3.2. Randomization

To perform a double blind test the images are not labeled during the visualization so neither the experts or the team can be biased by the images information (real, generated, nor its kind of generation). Real and generated images are randomized at each test run. Each biological expert evaluates the complete randomized dataset (280 images). The randomization and corresponding labels are stored in a .csv file which is only accessible for result analysis.

#### 3.3.3. Experts

The experts who evaluated the database are biologists from ERRMECe laboratory, EA1391, CY Cergy Paris University. The group of eight experts is composed of three men and five women who are either PhD students, research engineers or researchers. They all have an expertise in neuronal culture and microscopy acquisition. We do not allow duplicate evaluators across evaluation procedure. During each evaluation session the evaluator is physically isolated from the other participants, without knowledge of other experts responses or images labels.

#### 3.3.4. Evaluation software

**Figure 2:**
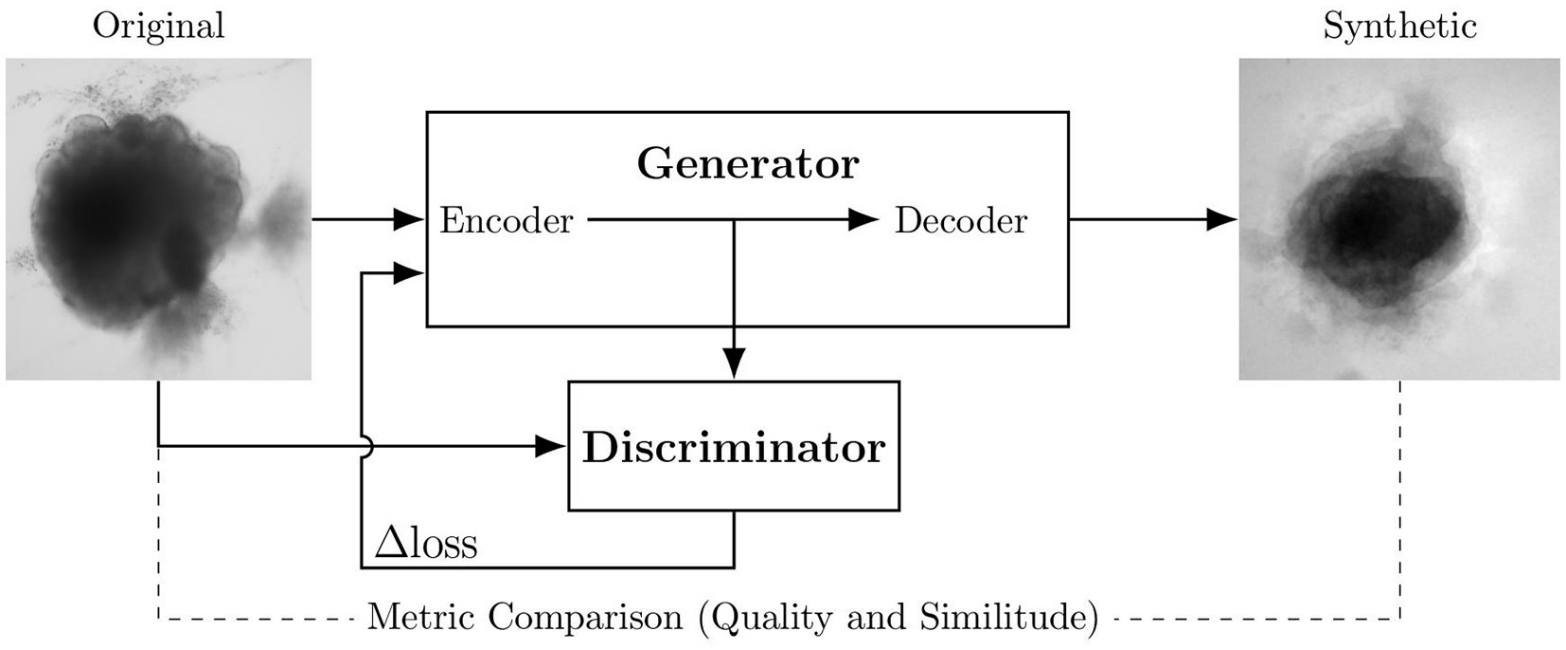
Experimental procedure of psychovisual evaluation. Left image represents a sample from original dataset and right image represents a specific loss optimized sample generated with an AAE. Screenshot of the software helping biological experts to decipher natural to non-natural content of brain organoid culture images. Eight biological experts test this procedure.

To help experts in their evaluation and to ensure consistency throughout the entire experiment, we built a dedicated software using Python 3.6, as shown Figure 2. The interface consists of an image displayed (250 × 250 pixels), a cursor with three buttons: *Real, Generated, Next* and the number of remaining images to classify. Keyboards shortcuts are available for *Real, Generated*, and *Next* buttons (respectively A, P, and Tab keys on an AZERTY keyboard) to facilitate the process. Images on the screen are updated each time *Next* is hit (or the corresponding keyboard shortcut). Clicks on *Next* are counted as a pass if not preceded by a *Real* or *Generated* click.

#### 3.3.5. Experimental protocol

An operator enters the expert name and the date and hour of the recording. All eight experts chose to use their own mouse with the experiment laptop. The protocol, consisting of a single session and including all 280 images (real and synthetic), is described in Figure 2. A pass is consider as an answer. The decision and answer time are saved at each click in a .csv file only accessible to analyze the results. The operator is present nearby to verify the smooth functioning of the experimental process and to capture any comments made by the experts. The list of questions the expert has to answer after the process is listed below:

- Why do you classify this generated image as a false? The operator shows the generated image with the longest hesitation.
- Why do you classify this original image as a false? The operator shows the original image with the longest hesitation, if this situation exists.
- What should we improve in future sessions?

We summarize the answers to these questions in the results section.

Each evaluation run produces two .csv files: One stores the randomization i.e. the order and label of each image presented. The second stores the experts' name and for each image present the answer time and the decision.

### 3.4. Analysis

The first analysis step consists in associating the randomization and the results files. We obtain, for each expert and for each image the decision and the decision time. The decision is then labeled as true positive (TP) or false negative (FN) for the original images and false positive (FP) or true negative (TN) for the synthetic images.

#### 3.4.1. Parameter calculation

It is then standard to calculate the error rate (ER), defined as the number of false decisions divided by the total number of decisions.


(16) $$\begin{array}{l} \text{ER}=\frac{\text{FP}+\text{FN}}{\text{FP}+\text{FN}+\text{TP}+\text{TN}} \end{array}$$

For the original images, this becomes:


(17) $$\begin{array}{l} {\text{ER}}_{\text{O}}=\frac{\text{FN}}{\text{FN}+\text{TP}} \end{array}$$

and for the synthetic images:


(18) $$\begin{array}{l} {\text{ER}}_{\text{G}}=\frac{\text{FP}}{\text{FP}+\text{TN}}. \end{array}$$

However, we wish to compare the proportion of synthetic images falsely labeled as true, with the proportion of original images label as true. We thus calculate the Positive Rate (PR) of the original images:


(19) $$\begin{array}{l} {\text{PR}}_{\text{O}}=1-{\text{ER}}_{\text{O}}=\frac{\text{TP}}{\text{FN}+\text{TP}}. \end{array}$$

For the synthetic images PR_G_ = ER_G_.

As a second parameter, we calculate the decision occurrence for each modality for each subgroup by a simple counting and render it in a % according to the total effective of a group of images.

We also evaluate the number of positive answers given by each expert as a count and the number of images given as a positive by zero expert, one expert, two experts etc. or the eight experts. Time decision and all these parameters are calculated between original and generated images, or between original and each modality of loss generation (BCE, BCE + L1, LS, Poisson, Wass., P. Wass.), globally or by each decision subgroups (FP, FN, TP, TN). All results are rendered as bargraphs representing variables (Time Decision in seconds or occurrence in % or error rates) according to one or many factors (group and subgroups of decision).

#### 3.4.2. Metrics vs. human decision

To verify if some metrics highlight the same loss as producing the most natural images as experts, we plot each metric values for each loss group by each decision factor modality (FP, FN, TP, TN). In the dot representations for each loss group, each metric is plotted according to the Normalized Error Rate NER with individuals decision time *t* for the FP and for FN modality:


(20) $$\begin{array}{l} \text{NER}=\frac{\text{FP}\times t_{\text{FP}}+\text{FN}\times t_{\text{FN}}}{\text{FP}+\text{FN}+\text{TP}+\text{TN}} \end{array}$$

where *t*_*FP*_ and *t*_*FN*_ are the average decision time respectively for FP and FN. In practice, this becomes, for the original images:


(21) $$\begin{array}{l} {\text{NER}}_{\text{O}}=\frac{\text{FN}\times t_{\text{FN}}}{\text{FN}+\text{TP}} \end{array}$$

and for the synthetic images:


(22) $$\begin{array}{l} {\text{NER}}_{\text{G}}=\frac{\text{FP}\times t_{\text{FP}}}{\text{FP}+\text{TN}}. \end{array}$$

To verify if a relation exists between a metric or a particular combination of metrics (*M*) and the decision or the time decision (*DT*), we calculate KL divergences on dimensional reduction results (Joyce, 2011).


(23) $$\begin{array}{l} KL=\underset{x\in X}{\sum }M_{x}\times \log\left(\frac{M_{x}}{DT_{x}}\right) \end{array}$$

We only represent here kl-divergence corrplots for each individual metric (and not metric combinations) for space considerations. We consider all possible metric combinations *C*:


(24) $$C=\left\{\left(\frac{n}{k}\right)|k\in N^{*},k\leq n\right\}$$

where *n* = 6 is the total number of metrics. The total number of metric combinations considered is thus 63.

We then calculate Pearson and Kendall correlations between metric combinations and KL-div results (for error rate and time decision) for original and synthetic groups. We show the Pearson correlation for the ten best metric combinations. The best representation of these results (error rate or time by KL divergence) are represented as a scatter plot.

#### 3.4.3. Statistical analysis

The normality is verified by a Shapiro and quantile to quantile graphics. We verify the homocedasticity in normal cases by a Bartlett test and in the case of non-normality by a Levene test. In case of a normality and homocedasticity, arametric tests are used (Anova), and non-parametrics tests otherwise (Kruskall–Wallis with a Tuckey *post hoc* test). Regression models are implemented to verify the interaction of factors (group and decision) on a specific variable (time or error rate or occurrence for instance). We use a *post-hoc* Holm test to compare two by two the effect of factors on variables after the regression.

We take an alpha risk at 5%. Correlation matrices are based on Pearson correlation tests.

### 3.5. Segmentation task

#### 3.5.1. Dataset

We first build a training dataset composed the 40 images from the original dataset and 40 images obtained by flip-flops, rotations, whitenings, or crops of these original images. This “clasical” dataset composes our baseline. We build five “psychovisual” training datasets where we replace part of the classically augmented images by synthetic images which are validated by 0, 2, 4, 6, or 8 experts. All datasets are composed of 80 images of which 40 original but the proportion of synthetic images decreases as the number of experts required to validate an image increases. Ground truth has been manually segmented with the ITK-SNAP software (Yushkevich et al., 2006).

#### 3.5.2. U-Net

Segmentation allows the extraction of an image content from its background. Various segmentation procedures exist but we have chosen U-Net which is widely used in the biomedical field (Ronneberger et al., 2015). U-Net has the advantage of working well for small training sets with data augmentation strategies, and has already been used for the ventricle segmentation of cleared brain organoids (Albanese et al., 2020).

#### 3.5.3. Training

**Figure 3:**
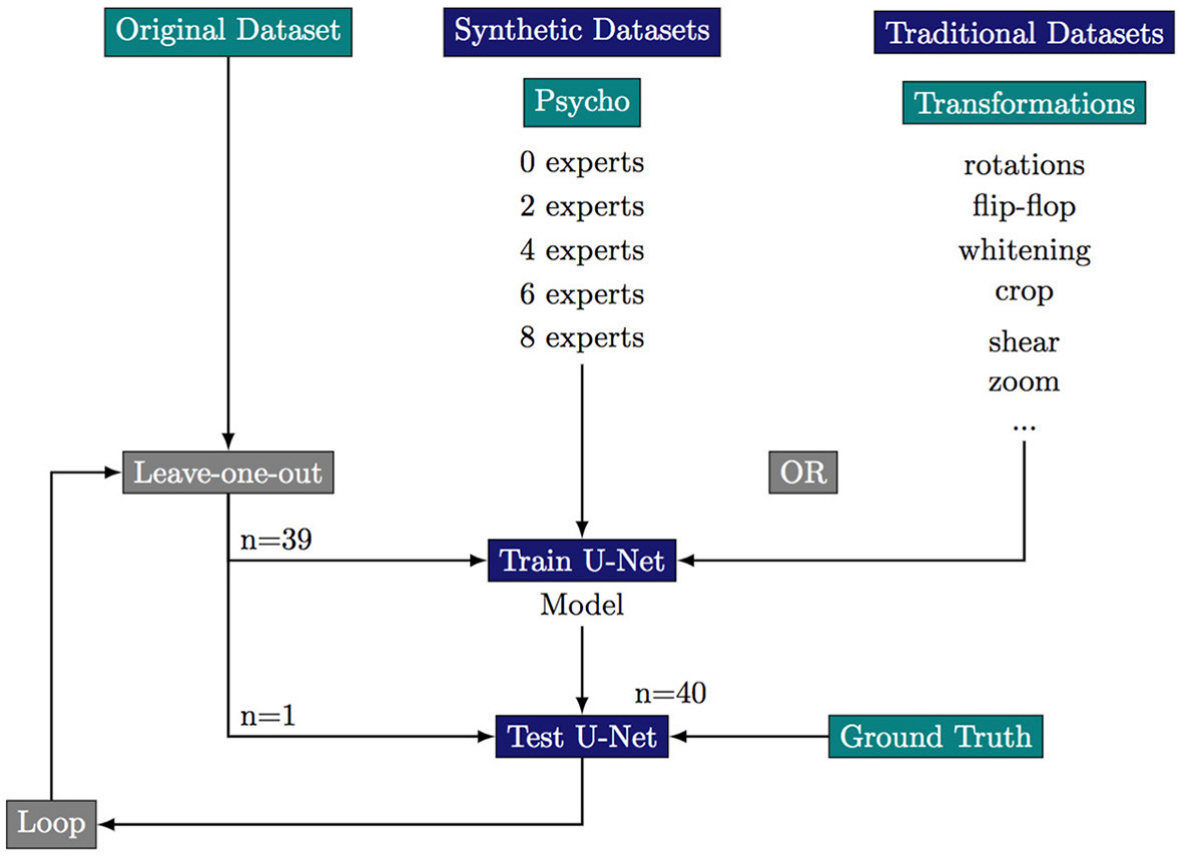
Experimental scheme of the leave-one-out strategy to test the effect of various data augmentation on the segmentation quality. ^***^If *p*-value ≤ 0.001.

To robustly evaluate the performance of the segmentation a these small datasets we use a leave-one-out strategy where we only test on the original images. This results in 40 training sessions per dataset. We stop the training at 1,000 epochs with an average time of training of more than 1 h for each leave-one-out loop (six cases of augmentations × 40 images = 240 h almost for the total training step). The summary of the leave-one-out strategy for every tested case is summarized in Figure 3.

#### 3.5.4. Comparison of segmentations

To compare ground truth cerebral organoid content segmentation (GT) and U-Net (u) ones in various conditions, mean Dice scores are calculated as:


(25) $$\begin{array}{l} Dice_{\left(GT,u\right)}=\frac{2|GT\cap u|}{\left|GT|+|u\right|} \end{array}$$

Thanks to the *TP*, *FP*, *TN*, and *FN* we could calculate the Accuracy, the Specificity, the Sensitivity, and the F1-score. The Accuracy is the ratio of true on the positives labels:


(26) $$\begin{array}{l} Accuracy=\frac{TP+TN}{TP+FP+TN+FN} \end{array}$$

The Sensitivity is the ratio between how much were correctly identified as positive to how much were actually positive:


(27) $$\begin{array}{l} Sensitivity=\frac{TP}{TP+FN} \end{array}$$

The Specificity is the ratio between how much were correctly identified as negative to how much were actually negatives:


(28) $$\begin{array}{l} Specificity=\frac{TN}{TN+FP} \end{array}$$

The Precision is the ratio between how much were correctly identified as positives to how much were actually labeled as positives:


(29) $$\begin{array}{l} Precision=\frac{TP}{TP+FP} \end{array}$$

The F1-Score allow to summarize the precision and the recall (Sensitivity) in an unique metric:


(30) $$\begin{array}{l} F1-Score=2*\frac{Precision*Sensitivity}{Precision+Sensitivity} \end{array}$$

#### 3.5.5. Visualization

To highlight real/false positive/negative segmentation we create a superimposed image composed by the ground truth and a sample of each segmentation resulting from the various trainings. We update the pixels values in lightpink the *FP* cerebral organoid segmentations and, in lightgreen the *FN*.

## 4. Results

We first present the metric evaluation of the synthetic images, then the results of the psychovisual evaluation of these synthetic images, and finally the correlations between the metrics and the psychovisual evaluation.

### 4.1. Qualitative evaluation

Table 1 shows three sample input images and three of the 40 synthetic images generated by each of the six AAE variations. Some of the generated samples are blurry and present a white imprint (BCE, BCE + L1, LS). Others show sharper edges and less visible imprints (Poisson, Wasserstein and P. Wass.). For this group of three losses, only a few of the generated images seem to be identical to a given input image. For example the Poisson loss produces three images which are a blurred version of the original. These networks do not suffer from mode collapse.

**Table 1:** Samples of original images and synthetic images generated by the AAE.

**Original**	**BCE**	**BCE + L1**	**LS**	**Poisson**	**Wasserstein**	**P. Wass**.
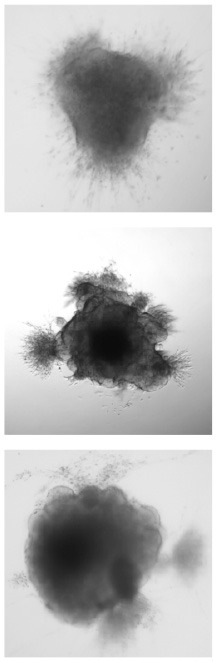	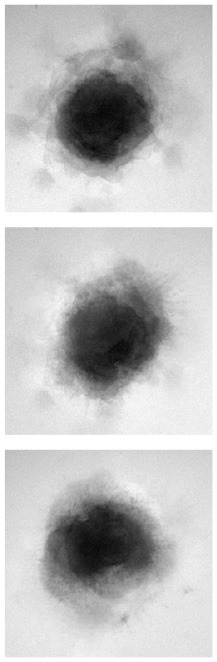	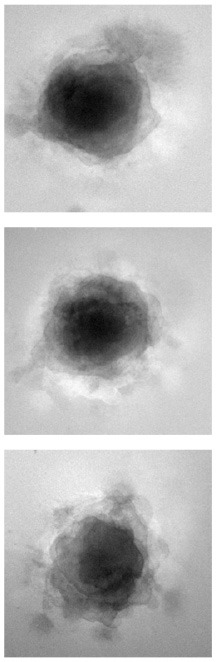	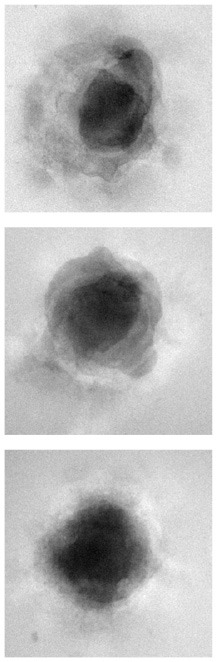	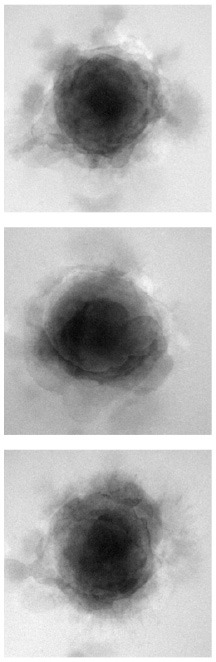	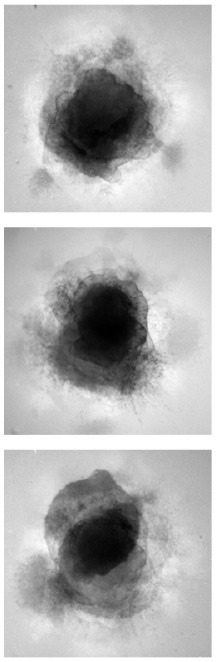	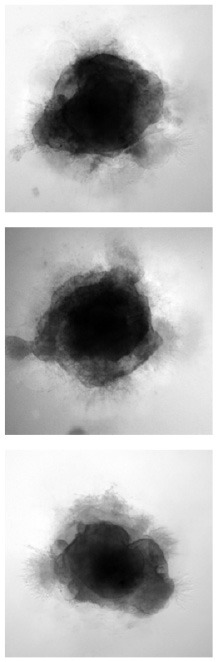

We show 3 of the 40 images for each group: original and per AAE loss variation.

### 4.2. Metric evaluation

To quantitatively confirm the visual analysis of the generated images, we calculate several metrics on both the original and synthetic images. These results are summarized in Table 2. This table presents the range of values given by the original images and the average value of each group of synthetic images (per loss optimization).

**Table 2:** Metric evaluation of AAEGAN brain organoid bright-field generated images.

**Metric**	**Best**	**Original**	**BCE**	**BCE + L1**	**LS**	**Poisson**	**Wass**.	**P. Wass**.
FID	Low	0.47–0.80	1.20	1.41	1.33	1.41	1.10	**0.82**
SSIM	High	0.65–0.71	**0.63**	0.62	0.60	**0.63**	0.62	0.50
UQM	High	0.63–0.87	0,83	0,83	**0.84**	**0.84**	0,83	0,82
MI	High	0.21–0.47	0.37	0.39	0.36	0.41	**0.46**	0.42
Blur	low	0.10–86.28	135.93	116.30	135.01	106.71	59.84	**59.00**
PSNR	Low	11.9–16.6	13.47	13.74	13.53	13.74	13.17	**12.86**
MSE	Low	93.25–106.23	103.13	103.35	104.01	103.33	103.11	**102.93**

We have calculated metrics on generated images from each AAE loss variations, with the BCE loss as the baseline. Scores outside the original range are in gray, best values are displayed in bold.

All six groups of synthetic images are within range of the original for the UQM, MI, PSNR, and MSE metrics. Images generated with a Poisson or LS loss have the highest UQM index. MI and MSE reach the best scores for the generated images using the Wasserstein losses. The FID and SSIM are out of range for all six groups of synthetic images. The average FID for the Perceptual Wasterstein loss is the closest to the original (0.82 vs. 0.80). Only the Wasserstein and Perceptual Wasserstein produce images that on average are within the range of the Blur metric for the original images.

Images generated with a Wasserstein and Perceptual Wasserstein loss are on average within the range of the original images for five out of seven metrics. Quantitatively, the Wasserstein and Perceptual Wasserstein networks generate images of a quality that most reassembles the original batch. In particular, the Perceptual Wasserstein loss generates the best results for four of the seven metrics. It appears to be the most appropriate loss optimization to generate cerebral organoid images with this AAE.

### 4.3. Psychovisual evaluation of synthetic images

**Figure 4:**
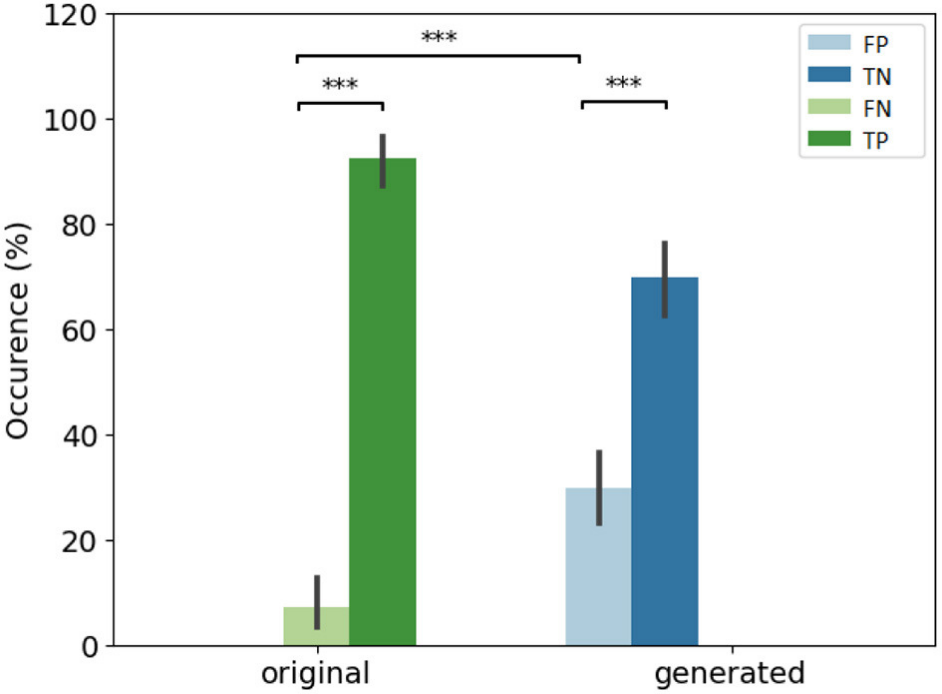
Overall view of decision per original and generated group. Error bars indicate the variability per expert. **(Left)** Error rate per original and generated group. The baseline (original) corresponds to positive rate PR. **(Right)** Occurrence of answers per original and generated group. ^*^If *p*-values ≤ 0.05, ^**^if *p*-value ≤ 0.01, and with ^***^if *p*-value ≤ 0.001.

In Figure 4, we compare the occurrence of each decision in percentages for *original* and *generated* groups. There is less misleading in original and generated group than right decisions. However, 30% of misleading is observed in the generated group. A misleading corresponds to a false positive answer.

**Figure 5:**
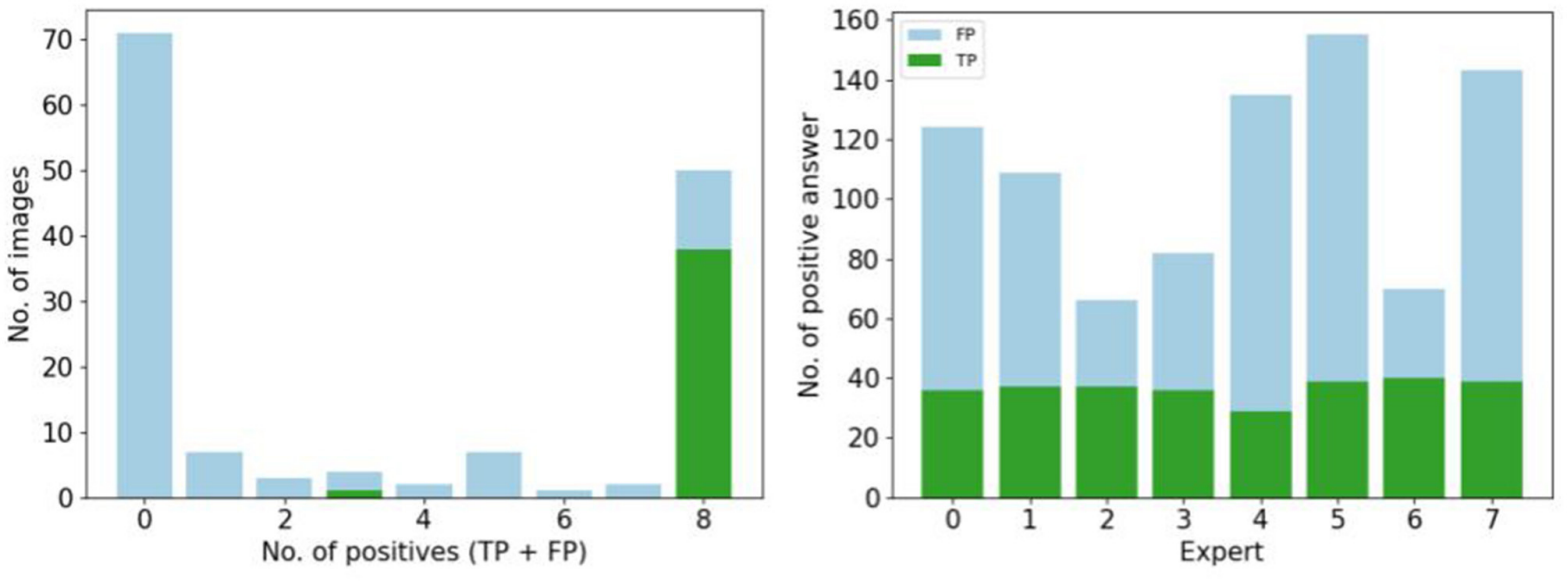
Study of positive answers. **(Left)** Number of images per number of positive answers. **(Right)** Number of positive answers by experts.

Figure 5 focuses on the images that are labeled as “natural” by the experts. We found the number of false positive selected images by all the participants is small (<20), and 30 images are selected by five participants. Almost 70 images are not selected at all by experts as natural (first column). To observe the number of false positive answer by each experts (see Figure 5). Three experts answer less positive answers than the others (less than the half of the visualized dataset). One expert considered over 150 images as natural.

**Figure 6:**
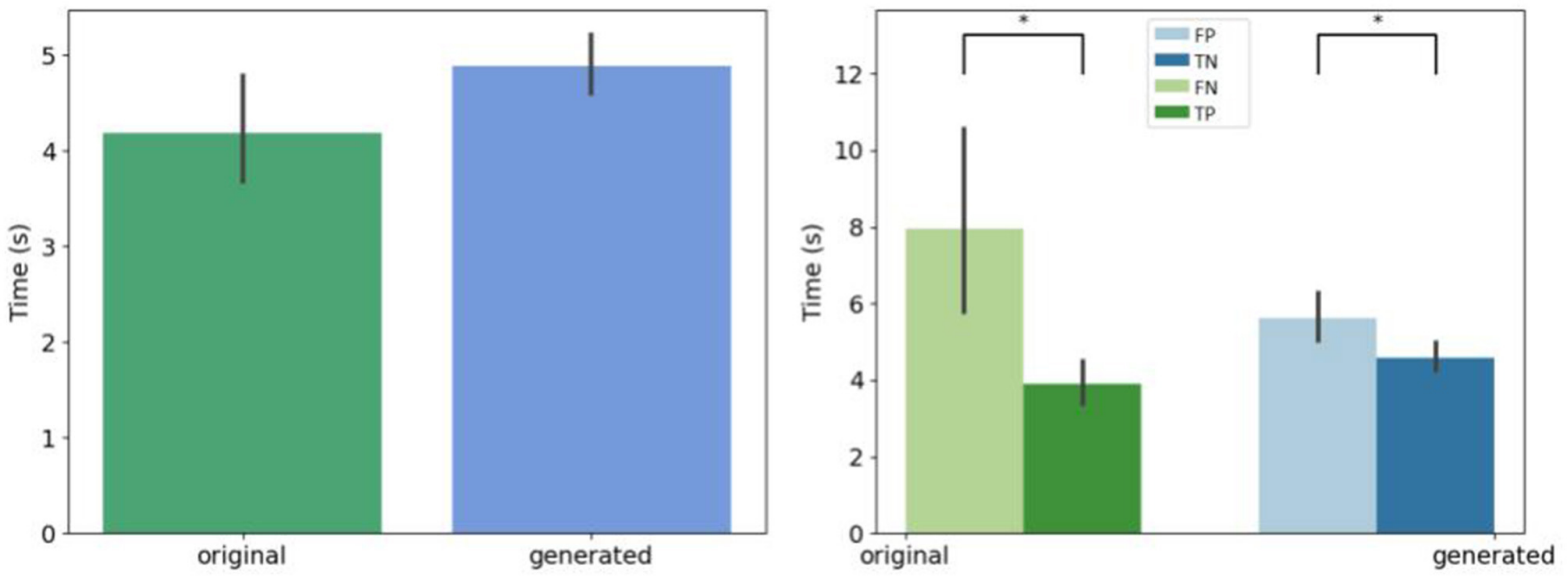
Mean decision time for all the experts. **(Left)** Per original and generated group. **(Right)** Per decision. ^*^If *p*-values ≤ 0.05.

We retrieve the decision time before the expert give an answer whatever the kind of generation, as shown Figure 6. Biological experts answer in the same time for generated and original images. However, the hesitation time is longer for false answers that for correct answers.

### 4.4. Feedback on the psychovisual procedure

Experts in cultures consider images as natural when an ovoid shape with neuroepithelial formation and some cell dispersion appears. When we ask experts why they classify a generated image as a synthetic, they answer the background contains an imprint, or superimposed contours or there is an artifact, or the image is too noisy, but they hesitate longer due to the possible content. They explain that they classify an original image as a synthetic because of a microscopic acquisition artifact, learned by one of the architectures, and reproduced on the worst synthetic images. They would like to have less images in a session, and a larger image on the screen.

### 4.5. Psychovisual evaluation of loss optimization

**Figure 7:**
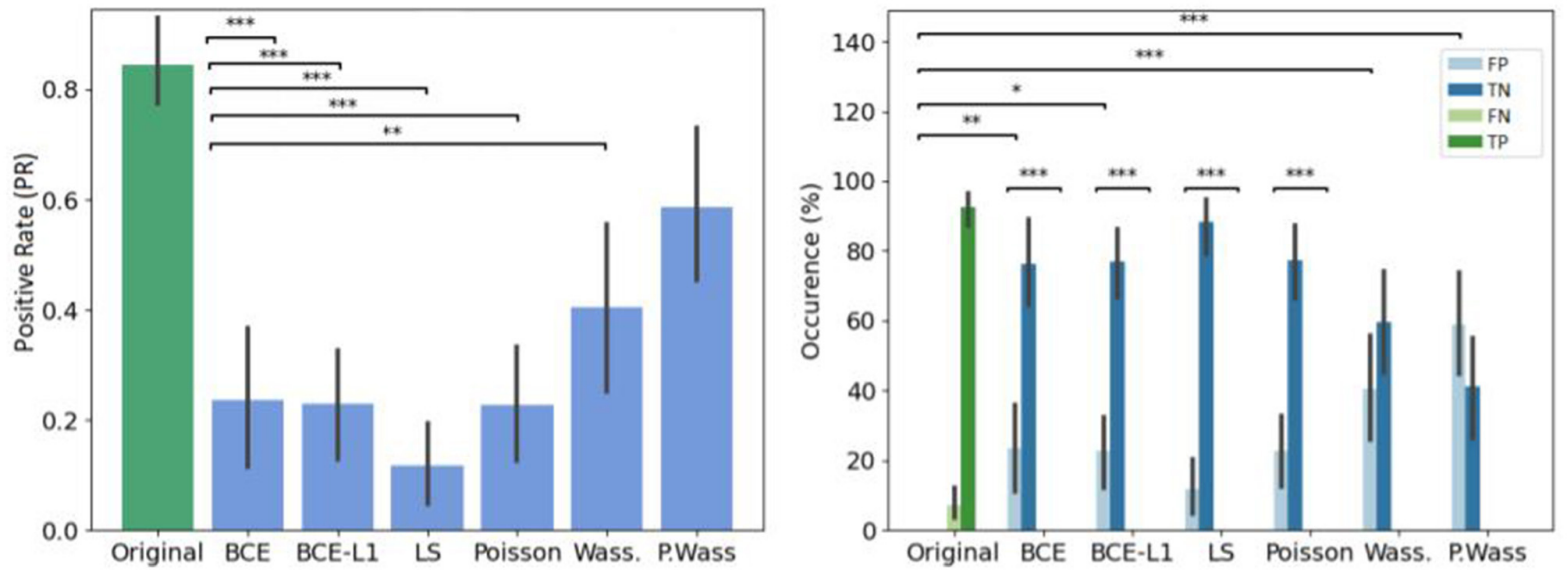
Overall results of decision per loss optimization groups. **(Left)** Effect of loss optimizations on the error rate. The baseline (original) corresponds to Positive rate (PR). **(Right)** Occurrence of answer per loss optimizations. ^*^If *p*-values ≤ 0.05, ^**^if *p*-value ≤ 0.01 and with ^***^if *p*-value ≤ 0.001.

The error rate is particularly higher in the Wass. and P. Wass groups than for the original one (see Figure 7 left). In the P. Wass case this high score is strengthen by the absence of statistical differences. These particular loss optimizations drive the experts to mislead and consider the images from these two groups as natural. In Figure 7 right, there is a difference between false positives of original and generated images from BCE, BCE+L1, Wass. and P. Wass. loss optimization. However, if we consider the intra-factor loss comparison, we can observe statistical differences between FP and TN of each for the BCE, BCE + L1, LS and Poisson loss rendering too small the proportion of misleading. There is no differences between these two occurrences decision for images generated by a Wass. or a P. Wass. loss showing 42% of FP and almost 60% of FP.

**Figure 8:**
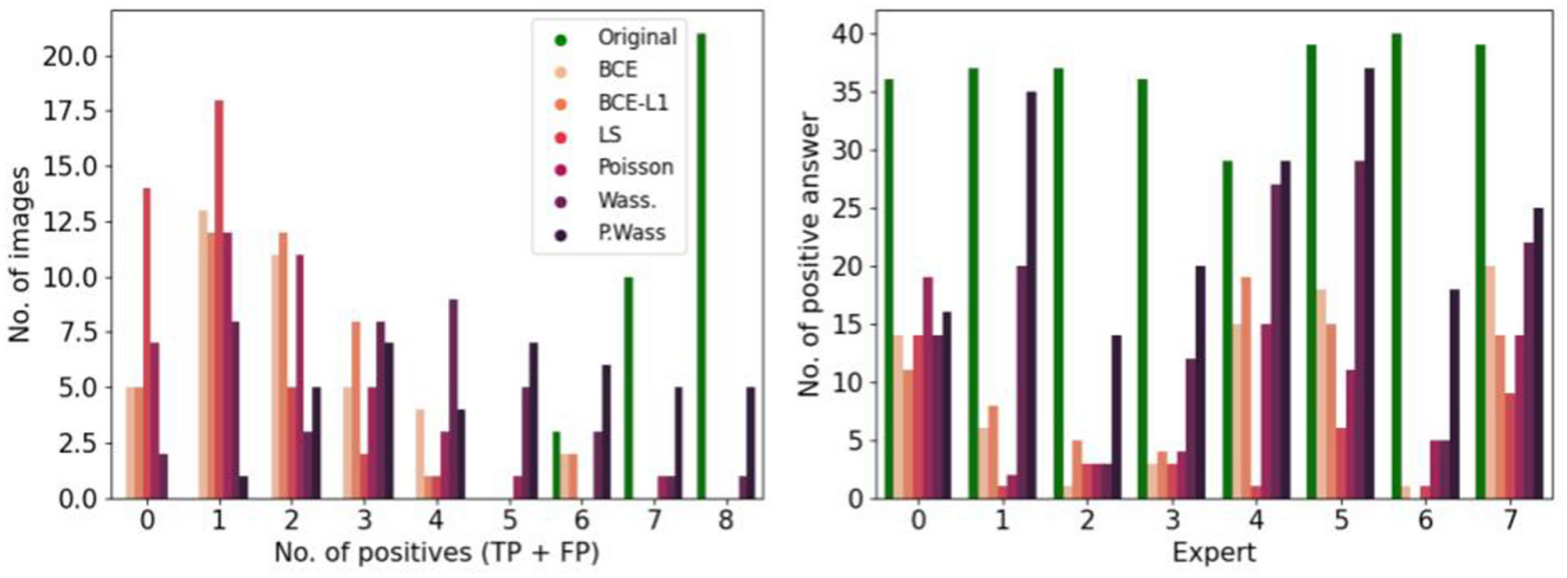
Study of positive answers per loss optimization. **(Left)** Number of images per loss optimization per number of positive answer. **(Right)** Number of positive per loss optimization for each expert.

To observe which group of images is the most selected as positive, we observe the number of image selected by group in the Figure 8 left, P. Wass. and Wass. images are selected as natural by the most of experts (a few Poisson, and a few BCE by seven experts and L1 + BCE). The same three experts as in Figure 5 only answer positive in most of the case for P. Wass. images (see Figure 8 right). Four experts answer more synthetic images but more even for P. Wass. Only one expert seems to answer identically for all synthetic group of images.

**Figure 9:**
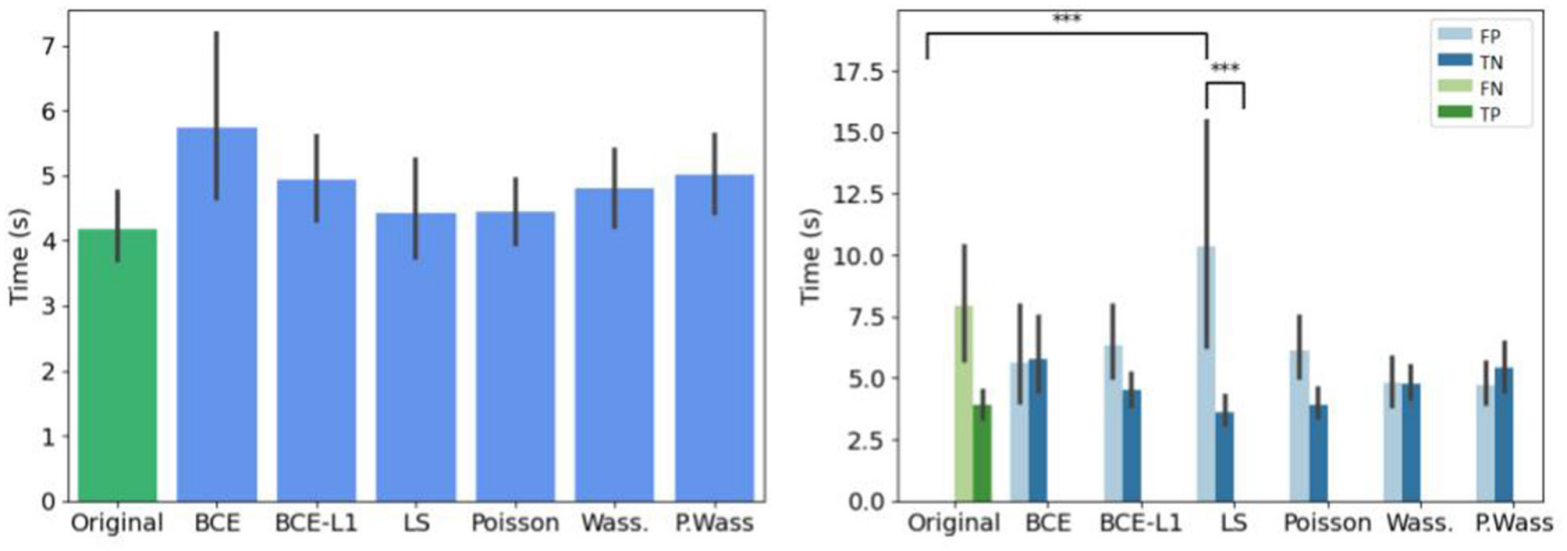
Mean decision time for per loss optimizations compared to the original input images. **(Left)** Globally. **(Right)** Per decision. ^*^If *p*-value ≤ 0.05, ^**^if *p*-value ≤ 0.01 and with ^***^if *p*-value ≤ 0.001.

If we do not consider the kind of decision, there is no difference of decision time per group of synthesis, (Figure 9 left). When we study the decision time per group, the experts take more time to answer only when they are confronted to synthetic images generated with a least square optimization (Figure 9 right).

### 4.6. Concordance of metrics and psychovisual evaluations

#### 4.6.1. Comparisons

After observing the psychovisual decisions by generated groups, we compare qualitative and similitude metrics to the previous results in order to verify if the same groups are selected, but also to verify if some metrics or combination of metrics can be used as a proxy to human psychovisual evaluation.

An overview of these results is given in Table 3 shows no differences between decision whatever the group of loss optimization or the calculated metric. FID is the highest for *Poisson* loss than for others groups whatever the kind of decision. BLUR metric is the highest for the decision with LS generated images. In term of SSIM, UQI indexes and PSNR, the decision reach the highest score for original images and no improvement is visible with generative methods. For MSE and MI the decision rate reach the highest score similarly for original and P. Wass. generated images. No differences are visible in term of decision with UQI.

**Table 3:** Average and standard deviation of five of metrics on original and synthetic images per psychovisual decision (true or false) and per loss optimization.

	**Decision**	**Count**	**Blur**		**SSIM**		**PNSR**		**MSE**		**MI**		**UQI**	
			**avg**.	**σ**	**avg**.	**σ**	**avg**.	**σ**	**avg**.	**σ**	**avg**.	**σ**	**avg**.	**σ**
**Positive**								
Original	TP	297	64.15	50.28	**0.78**	0.17	**42**	41.49	2973	3163	0.90	0.46	**0.86**	0.12
BCE	FP	79	100.22	15.40	0.62	0.06	13.52	3.20	3713BCEL1	FP	77	104.16	38.89	0.61	0.07	13.54	2.94	3549	2320	0.82	0.13	0.83	0.09
LS	FP	39	**155**	44.78	0.59	0.07	13.77	3.00	3403	2276	0.78	0.11	0.84	0.09
Poisson	FP	74	93.17	22.76	0.63	0.06	13.86	2.84	3275	2139	0.86	0.14	0.84	0.09
Wass.	FP	134	61.47	18.25	0.61	0.06	13.33	2.38	3499	1980	0.90	0.14	0.83	0.07
P. Wass.	FP	197	47.19	17.13	0.63	0.07	12.48	2.44	**4272**	2389	**0.95**	0.17	0.80	0.07
**Negative**								
Original	FN	23	44.93	25.36	0.71	0.01	10.10	0.90	6418	1326	0.88	0.23	0.78	0.04
BCE	TN	241	105.01	20.95	0.62	0.06	13.57	3.16	3647	2555	0.85	0.13	0.83	0.10
BCEL1	TN	243	110.43	39.30	0.60	0.07	13.60	2.87	3477	2248	0.81	0.13	0.83	0.09
LS	TN	281	**156**	44.21	0.58	0.06	13.79	2.80	3307	2118	0.78	0.11	**0.84**	0.09
Poisson	TN	246	106.12	25.34	0.62	0.06	**13.86**	2.77	3248	2090	0.84	0.13	**0.84**	0.09
Wass.	TN	186	56.96	17.18	**0.63**	0.07	13.41	2.58	3510	2116	0.90	0.13	0.83	0.07
P. Wass.	TN	123	41.22	14.04	**0.63**	0.07	12.69	2.55	**4123**	2414	**0.95**	0.16	0.80	0.075

Best values are displayed in bold.

#### 4.6.2. Correlations

**Figure 10:**
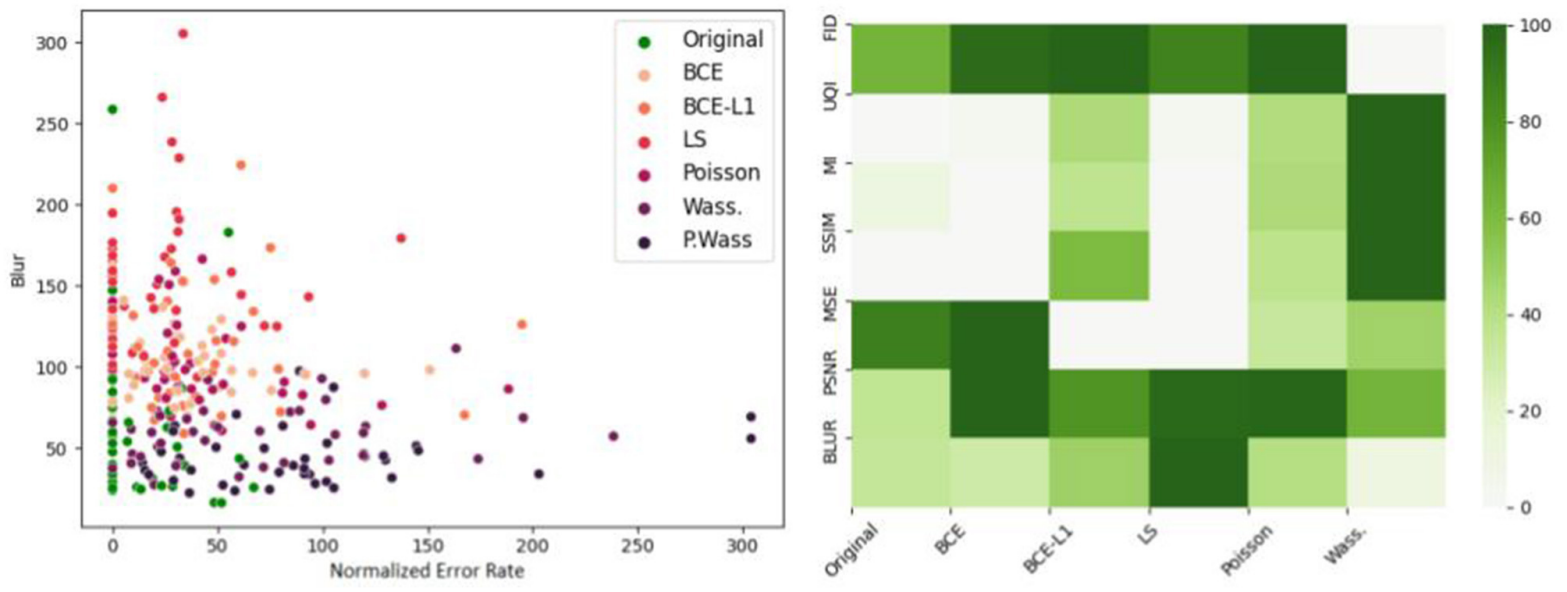
Comparison of error rate per optimization losses and per metrics. **(Left)** Blur score with respect to the normalized error rate for all 280 images grouped by optimization loss. **(Right)** KL divergence between the P. Wass group and all other groups for all metrics. Dark green represents a strong divergence between. Groups with a small KL divergence groups with respect to P. Wass. are white.

To identify the metrics which best correspond to the psychovisual evaluation, we plot metrics against error rate in Figure 10. We show the Blur scatterplot as an example of point representations. In this graphic, we observe that the green color points (*Original*) are near the darker-purple ones (P. Wass.). Figure 10 also represents the KL divergence between the P. Wass group and all other groups for all metrics. If we look at the first column, P. Wass and Original images are closest according to SSIM, MI and UQI. These metrics are good candidates to build a metric that mimics psychovisual evaluations.

**Figure 11:**
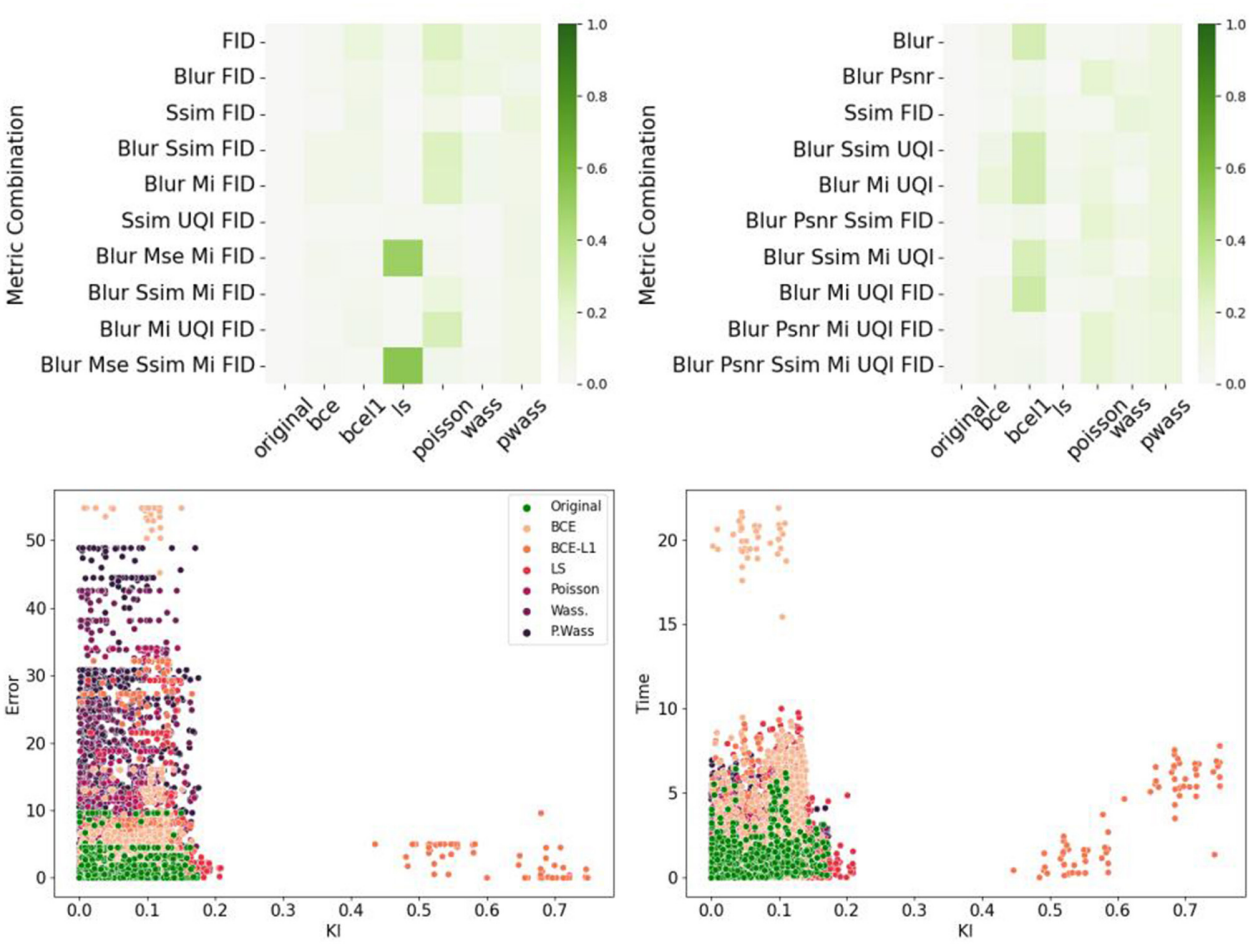
Correlations between metrics and psychovisual assessment on all 240 synthetic images. **(Left)** Error rate. **(Right)** Hesitation time. **(Top)** Correlation matrix of the ten best combinations. **(Bottom)** Example of error rate (resp. hesitation time) over KL divergence for a given metric combination: **(Left)** Blur and FID. **(Right)** Blur SSIM MI and UQI.

Figure 11 summarizes the correlations between the psychovisual assertion and the hestitation time or error rate according to each metric or chosen metric combinations. The main result is the absence of correlation for single metrics, however, combination with a Blur metric, FID (for the error rate correlations) and SSIM-FID-MI (for the time correlation) render the highest results in Figure 11 top left. The same result is represented in Figure 11 top right. To observe the group representation between the Error rate or the Time and the KL divergence of points represented for these two combinations (Blur-FID and Blur-SSIM-FID-MI) (see Figure 11 bottom right and left). The LS and BCE group are far from the others point representations. P. Wass. group is superimposing the original one with Wass. Others groups are not distinguishable, however, there are at the peripheral zone of the perceptual-original amount. The KL divergence representation with respect to the error rate of these two metric combinations is given in the bottom part of Figure 11.

### 4.7. Influence of psycho-validated images on a segmentation task

Then the second task is to verify the interest of using synthetic images which have been validated by 0, 2, 4, 6, or 8 biological experts to train a segmentation task.

#### 4.7.1. Qualitative results

To observe the quality of the segmentation, we show a ground truth segmentation performed with the ITK-SNAP software, and automatic segmentations performed with a U-Net architecture with various data augmentation strategies, see vignettes in Table 4. The “0 experts” group corresponds to training the segmentation with images that have been selected by none of the experts. In the others training images are previously selected by 2, 4, 6, and 8 experts. We observe less false positive regions (in pink) and false negative regions(in green) if the segmentation is performed after training on a dataset containing images validated by 6 or 8 experts. If synthetic images selected by six or more experts are used for training, we observe almost no errors on the segmentation.

**Table 4:** Segmentation results after training on a varying number of synthetic images.

	**GT**	**Classical**	**No. of experts validating the synthetic images**				
			**0**	**2**	**4**	**6**	**8**
No. of synthetic images	–	0	33	41	22	16	14
Sample result	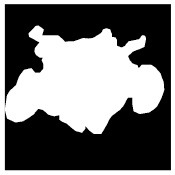	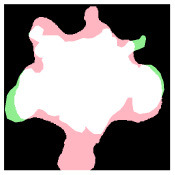	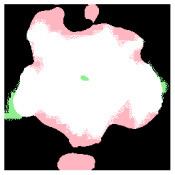	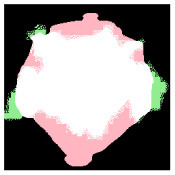	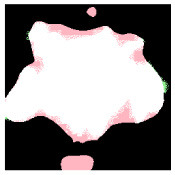	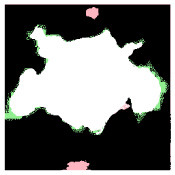	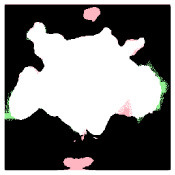
					Dice			1.0	0.80	0.62	0.59	0.61	0.67	**0.82**
Accuracy	1.0	0.77	0.63	0.61	0.68	0.62	**0.93**
Sensitivity	1.0	0.94	0.96	**0.97**	0.87	0.83	0.91
Specificity	1.0	0.92	0.50	0.48	0.60	0.56	**0.94**
F1-score	1.0	0.84	0.64	0.61	0.64	0.59	**0.87**

Seventy-nine images are used for training of which 39 are from the original data set. The remaining 40 are a combination of classic transformations and synthetic images. Tests performed with no synthetic images (classical) are compared to those performed with synthetic images validated with an increasing number of experts (0 to 8). Best values per metric are displayed in bold.

#### 4.7.2. Quantitative results

Table 4 summarizes the metrics used to compare the ground truth segmentation with the segmentation performed by U-net trained on data augmented with classic strategies or on a varying portion of synthetic images. The segmentation is better when the network is trained with images validated by 8 experts, with higher levels of Dice, Accuracy, Sensibility and F1-score. The highest sensitivity is reach by the group trained on 41 synthetic images validated by two experts and by the group trained on images validated by no experts. However, the corresponding specificity is very low.

## 5. Discussion

In this part we present to our knowledge the first psychovisual and metric evaluation comparison of Loss optimized generative adversarial network of brain organoid bright-field images. This study helps at validating most natural images generated by various AAE loss optimizations. We also contribute to strengthen metric evaluation by highlighting some images from optimized generated adversarial network to be perceived by Human biological expert as natural microscopic images: with a P. Wass. loss perception neurobiologists are misled 60% of the time and 40% by Wass. They take more time to answer when they are misled. We compare human and metric evaluation and found mutual information to be the most related to their decision metric, although no correlation appeared in our experiment for single metrics, but only for combinations including blur. Using synthetic images validated by an increasing number of experts to train a segmentation network increases the accuracy of the segmentation with respect to classic augmentation strategies, even if the proportion of synthetic images decreases.

Synthetic images generated with AAE are coherent with original dataset and thus whatever the kind of loss optimization. The generation of brain organoid images with others architectures does not improve the synthesize in term of quality or similitude according to Brémond Martin et al. (2022a), whereas it seems the case with loss optimization. The P. Wass. loss optimization of AAE performs best according to metrics. Other loss optimizations show also high similitude, though with a lower quality. In this context, we plan to explore what type of information each loss brings during the image generation. We aim at trying others embedded losses (already used for segmentation tasks) during the generative process based upon high level prior like object shape, size topology or inter-regions constraints (El Jurdi et al., 2021). These losses could be used on condition that the morphological development of CO is better characterized.

Biomedical experts select around 40% of synthetic images as natural compared to the original dataset. Thus, the generation by AAE networks generate a large part of realistic images such as the background of bright-field acquisition or, their content. The non-selected images where considered sometimes as non-natural due to some artifacts reproduced in some of them, or by a superposition of contours. Nevertheless, the selected images can help train a DL segmentation network.

A first argument of the strong validation of the selected images as natural is the time to take a decision (Shaffrey et al., 2002). If the time to answer natural for a generated group corresponds to the time to answer natural or original images, we could consider these two groups are perceived as similar. We found no differences between original and generated images and thus whatever the kind of loss optimization used to produce them. So they are not doubting when they classify an image as natural or generated. However psychovisual evaluation shows an increase of decision time before answering when they answer as false positive (depending of the loss optimization) or false negative. This behavior is specifically shown from a Least Square Loss Optimization generated images considered as natural. When we ask participants why they have doubt on a particular image, they answer that it was linked with some acquisition artifact learned by the generated process and found on a lot of images (a bunch of cells) or, by a blurry contour which could be due to the acquisition in the case of original images (Ali et al., 2022). For false negative answers, they only said that the artifact acquisition is also present (and they thought it was a generated). In the future, we think a pre-process image treatment has to be done on images to correct the acquisition artifact before the generative process, to avoid these false negative in the psychovisual evaluation or, to add a component in the generative network to avoid these artifacts (Galteri et al., 2017; Ali et al., 2022).

Only 15 images are selected by all the experts as natural. Five experts select over 100 images as natural and three <80. This study raises a question: could we use images considered by only five experts as natural in a training step? Thus we thought we need to increase the number of biological experts to overcome future studies in order to be more precise on the number of images considered as natural. We could also analyze the answers by the field of expertise of biological experts too (separate those who made only culture or only microscopic acquisition from those working in both fields).

Nonetheless, human Psychovisual experts choose in majority images from generative adversarial network as natural if they are from Wass. and P. Wass. loss and, a few BCE, BCE-L1. These two first kinds of generation are also highlighted by most of the metrics in an other study to have the better quality and to be the most similar to original images (Brémond Martin et al., 2022a). Thus, the psychovisual evaluation strengthen the choice of the use of these two and particularly the perceptual one in generative process. We can now confirm the idea that the regulation term of the Wass. distance between two images (Kupyn et al., 2018) could improve the learning of the pattern or characteristics of brain organoids in images and contribute to generate more natural images in term of content and aspects. In future studies we will remove the images that did not dupe the experts to only train on human-validated images. We would like to see if this increases the segmentation accuracy and study the impact on the morphological characterization.

However, the few BCE and BCE-L1 images selected as natural by psychovisual experts could maybe have also a great interest whereas the metric are not pointing them as natural images (Brémond Martin et al., 2022a). As we know the use of metric is still controversial for the GAN evaluation as they are measuring similitude and quality (Borji, 2019). Here, we could not highlight a strong correlation between the use of certain metrics and the decision to reject or not a generated image as natural. To correlate a metric and psychovisual evaluation instead of a binary answer “natural” or “not natural,” some authors use a graduation scale (Pedersen and Hardeberg, 2012; Pedersen, 2015). This approach could be tested in future studies.

No metric used in this study could replace a human perceptual evaluation to decipher the naturality of an image generated. There is a certain link with FID, BLUR or MI and the group and MI with the mean decision but it remains weak. We could only say that similitude and referenced-bases metrics are more linked to the decision than qualitative metrics and non-reference-based metrics. And when we compare metrics with decisions some patterns appear according to the kind of loss optimization. The use or not of a measure to decipher natural generated examples is an issue recently discussed (Borji, 2019). To compare fairly images generated by various optimized models, there is no consensus for a use of a particular metric. In other fields such metric comparisons highlight a wavelet structural similitude index WSSI, a metric which based upon SSIM but less complex and more accurate in term of quality assessment (Rezazadeh and Coulombe, 2009; Pedersen, 2015). However, we do not want an identical image but one just resembling as a natural one. This could explain these metrics are not well designed for the GAN specific evaluation when they are considered alone. This study comparing the overall psycho-visual evaluation and seven metrics is one of the pioneer work which could contribute to help at pointing a metric of “natural,” and it failed partially.

Based upon our KL divergence maps, we suggest that a combination of metrics which best represents the psychovisual evaluation decision (BLUR, SSIM, MI, UQI) could be used as a substitue for a human psychovisual evaluation which is time consuming. Nevertheless, this work on metric combinations replacing a psychovisual evaluations need to be further studied. In other fields the combination of metrics help at pointing out some results in term of quality or similitude (Yao et al., 2005; Pedersen and Hardeberg, 2012; Okarma et al., 2021). An other idea could also to use non-reference quality metrics combinations (Rubel et al., 2022). Some authors tries also to implement directly a discriminator of generative adversarial networks based upon human perception, this could be a solution if it is not time consuming (Fujii et al., 2020; Arnout et al., 2021). It is not the case in this study, for us, an important task is to found an appropriate metric for highlighting “the naturality” of the image and replacing the psycho-visual evaluation. An idea is to test psycho-metrics instead of classical similitude or quality metrics such as Hype from Zhou et al. (2019) which is an alternative of *FID* from Heusel et al. (2017), or implementing the GFI quality assessment created by Tian et al. (2022).

The more experts validate a portion of synthetic images in the dataset, the better the segmentation quality. This suggests that if more experts are available to select images, and strengthen the naturality of the synthetic dataset used during the training, it could improve the accuracy of the segmentation results. However, even if the psychovalidation of certain synthetic images allows us to improve the segmentation, this method is still subjective. It could include biases of the experts about the model, its configuration, and the project objective. It requires knowledge of which images are considered as natural and which ones are not for the target domain, so the number of experts available in the field diminished while our task required more experts. It is limited to the number of images that can be reviewed in a reasonable time (Booij et al., 2019).

Validation by six experts is the minimum to improve the segmentation, but quantitative analysis show us, eight experts validation is the minimum due to the equilibrium state between the specificity and the sensibility. The performance of human judges is not fixed and can improve over time, other articles choose a validation by 15 experts for instance which is not possible in our biomedical context which requires experts in the field (Denton et al., 2015; Salimans et al., 2016b).

We could also apply this psychovisual evaluation on others datasets to attempt to answer more specifically to the metric replacement. We thought about noise optimized generated images of brain organoids with an AAE for the same aim (Brémond Martin et al., 2022b). It could be interesting to observe if with a noise injection, similar to the bright-field acquisition images, generated images are more perceive as natural even if metrics are not pointing a particular kind of noise. Indeed, qualitative and similitude metrics point out Gaussian noise and shot noise injection. But as said previously, this could maybe only due to the metric choice (Borji, 2019). An analysis of Psychovisual evaluation could maybe help at highlighting a combination of metrics. In this future study, it could be also interesting to observe for example the microscopic experience of the Biological expert as a new criterion. A larger application of this methodology could be made on others kind of generation (such as on GAN, Goodfellow et al., 2014 or DCGAN, Radford et al., 2015) and maybe help at pointing out the best GAN model for brain organoid generation used during a training segmentation task.

In this study we use a unbalanced dataset with more synthetic images than original. Nevertheless, biological experts do not know the number of real or synthetic images which render it unbiased. In future studies, we need to obtain and use more original images in order to re-equilibrate. We use a software created specifically for the psychovisual task for brain organoid images. The software needs to be updated due to some limitations. We have to realize batch process with pauses to limit the tiredness of biological experts similar to others psychovisual evaluations (Shaffrey et al., 2002). We have to add also a cursor with a score instead of a button to estimate a natural range in future studies and facilitate correlations studies (Tian et al., 2022). The size of the image of the screen has to be increase but not for all the participants. Apart from these updates, the use of the software is simple and practical according to their feedback.

To strengthen our statistical analysis we should increase the number of biological experts. However, it could include biases of the experts: it requires knowledge of which image is considered as natural and which one is not for the target domain, so the number of experts available in the field is diminished while our task required more experts. The performance of human judges is not fixed and can improve over time, other articles choose a validation by 15 experts for instance which is not possible in our biomedical context which requires experts in the field (Denton et al., 2015; Salimans et al., 2016a). Moreover, psychovisual evaluation is limited to the number of images that can be reviewed in a reasonable time (Borji, 2019). The tradeoff between the number of synthetic images used to train a network and the number of validating experts could be further explored.

## 6. Conclusion

In this study psycho-visual evaluations allow us to:

- Validate some synthetic image generated from loss optimization of generative brain organoid images with an AAE in term of decision time and decision.
- Describe the quality and similitude of the synthetic images with the original dataset by a metric validation.
- Verify if some synthetic images could be considered as natural by psychovisual expert decision.
- Compare psychovisual and metric evaluations.
- Paves the way to finding a metric or a metric combination that mimics psychovisual evaluations.
- Show the interest of selecting images validated by the highest number of experts in a data augmentation strategy for a segmentation task.

This selected images could be use in the training phase of a segmentation task in order to help at their morphological development characterization for instance. We also need to evaluate psychovisually noise injected optimized synthesized images.

In future studies we suggest a combination of metrics or a perceptual metric could maybe help at replacing the psycho-visual assessment which is time consuming. Such methodology could be used for others brain organoid data-sets generated with a generative adversarial network.

## Data availability statement

The raw data supporting the conclusions of this article will be made available by the authors, without undue reservation.

## Author contributions

This article was an idea of CB-M, CS-C, CC, and AH. The literature search and data analysis was performed by CB-M. Graphics tables and figures were conceived by CB-M and CS-C. The first draft was written by CB-M while CS-C, CC, and AH critically revised the work. All authors contributed to the article and approved the submitted version.
